# Efficacy of Nanofiber Sheets Incorporating Oxaliplatin in Gastrointestinal Cancer Xenograft Models

**DOI:** 10.3390/nano15191524

**Published:** 2025-10-05

**Authors:** Fusao Sumiyama, Hoang Hai Duong, Hideyuki Matsushima, Kosuke Matsui, Terufumi Yoshida, Hidekazu Yamamoto, Hisashi Kosaka, Mitsugu Sekimoto, Van Khanh Nguyen, Thanh Tung Lai, Takuya Ohigashi, Tomoya O. Akama, Kengo Yoshii, Emiho Oe, Nanami Fujisawa, Mitsuhiro Ebara, Masaki Kaibori

**Affiliations:** 1 Department of Hepatobiliary Surgery, Kansai Medical University, Osaka 573-1191, Japan; sumiyama.fus@kmu.ac.jp (F.S.); m241201@med.kmu.ac.jp (H.H.D.); matsushh@hirakata.kmu.ac.jp (H.M.); matsuik@hirakata.kmu.ac.jp (K.M.); yamhidek@hirakata.kmu.ac.jp (H.Y.); kosakahi@hirakata.kmu.ac.jp (H.K.); m231204@med.kmu.ac.jp (V.K.N.); laithanhtung@hmu.edu.vn (T.T.L.); ohigasht@hirakata.kmu.ac.jp (T.O.); 2 Department of Gastrointestinal Hepatobiliary Surgery, Thainguyen National Hospital, Thainguyen 240000, Vietnam; 3 Department of Colorectal Surgery, Kansai Medical University, Osaka 573-1191, Japan; yoshiter@hirakata.kmu.ac.jp; 4 Conjoint Gastroenterology Laboratory, QIMR Berghofer Medical Research Institute, Herston, QLD 4006, Australia; 5 Department of Surgery, Kansai Medical University, Osaka 573-1191, Japan; m.sekimoto@minoh-hp.jp; 6 Internal Gastroenterology Department, VNU University of Medicine and Pharmacy, Hanoi 100000, Vietnam; 7 Department of Surgery, Hanoi Medical University, Hanoi 100000, Vietnam; 8 Department of Pharmacology, Kansai Medical University, Osaka 573-1191, Japan; akamat@hirakata.kmu.ac.jp; 9 Department of Mathematics and Statistics in Medical Sciences, Kyoto Prefectural University of Medicine, Kyoto 602-0841, Japan; yoshii-k@koto.kpu-m.ac.jp; 10 Research Center for Macromolecules and Biomaterials, National Institute for Materials Science (NIMS), Ibaraki 305-0047, Japan; oee@nissanchem.co.jp (E.O.); s2230101@s.tsukuba.ac.jp (N.F.); ebara.mitsuhiro@nims.go.jp (M.E.)

**Keywords:** oxaliplatin, polycaprolactone, electrospun nanofiber sheet, MKN 45, COLO 205

## Abstract

Oxaliplatin is an anticancer drug used to treat colorectal and gastric cancers. In many cases, chemotherapy is discontinued due to adverse events caused by anticancer drugs. To address this challenge, we developed a sustained-release drug delivery system using polycaprolactone sheets embedded with oxaliplatin (oxaliplatin sheets) and evaluated their therapeutic potential in murine models of colon and gastric cancers. Antitumor efficacy was compared with conventional intraperitoneal administration by monitoring tumor volume, body weight, and systemic oxaliplatin concentrations over 21 days, along with histopathological assessment of tumors and hepatic tissue. Oxaliplatin sheets demonstrated superior tumor suppression, significantly reduced Ki-67 positivity, and mitotic indices. Additionally, antitumor effects and blood oxaliplatin levels were consistent regardless of implantation site. Notably, oxaliplatin sheets significantly decreased weight loss compared with intraperitoneal administration. In our analysis of liver pathology, we found that hepatic sinusoidal obstruction and hepatocellular degeneration were significantly increased after intraperitoneal administration compared with untreated mice and mice treated with oxaliplatin sheets. Furthermore, treatment with oxaliplatin sheets improved survival. Thus, our oxaliplatin sheets exhibited effective tumor control and reduced side effects, indicating their potential as a promising treatment for advanced gastric and colorectal cancers.

## 1. Introduction

Colorectal and gastric cancers are the most common malignancies in the world. Among all types of cancer, colorectal cancer ranks third in incidence and second in mortality, whereas gastric cancer ranks fifth in incidence and fourth in mortality [1]. Although these cancers are treated with a variety of therapies worldwide, advanced colorectal and gastric cancers are both associated with poor prognoses. Management of colorectal and gastric cancers typically involves a multimodal approach, including surgery, systemic chemotherapy, and radiotherapy. For most patients with unresectable advanced or recurrent disease that has metastasized, systemic chemotherapy remains the standard of care.

Oxaliplatin is the main anticancer drug used in chemotherapy regimens for gastric and colorectal cancers. In Japan, the United States of America, and Europe, regimens such as SOX (S-1 and oxaliplatin), CapeOX (capecitabine and oxaliplatin), and FOLFOX (folinic acid, 5-fluorouracil, and oxaliplatin) are used to treat metastatic gastric cancer [2,3,4], and regimens combining FOLFOX, FOLFOXIRI (folinic acid, 5-fluorouracil, oxaliplatin, and irinotecan), and molecular-targeted agents are used to treat metastatic colorectal cancer [5,6,7].

Oxaliplatin treatment is known to cause several adverse events, including pancytopenia, vomiting, diarrhea, fatigue, weight loss, and peripheral neuropathy. Furthermore, oxaliplatin can result in sinusoidal obstructive syndrome (SOS) [8], a liver injury that is thought to be primarily caused by sinusoidal obstruction due to damage to the sinusoidal endothelium. Most liver injuries due to SOS are mild [9], but cases of liver failure and rupture of varicose veins have also been reported [10,11]. Although advances in chemotherapy have improved survival rates, these adverse events often prevent patients from continuing effective chemotherapy, forcing dose reductions or regimen changes.

Research on drug delivery systems (DDSs) in cancer chemotherapy has been attracting attention; this is because DDSs are expected to improve tumor targeting, enhance drug stability in the body, and reduce side effects caused by these factors. In particular, nanoparticle drug carriers are expected to improve the blood stability of anticancer drugs and tumor site-specific treatment via the enhanced permeability and retention (EPR) effect [12,13]. Other DDS technology-based cancer treatment strategies, such as metronomic chemotherapy and dose-dense therapy, are expected to be used to prevent cancer cell growth through the continuous administration of anticancer drugs over long periods of time.

We have designed a nanofiber platform as a DDS carrier. Due to their large surface area, nanofibers can release a constant concentration of drug over a long period of time [14,15]. In addition, nanofibers can essentially encapsulate many drugs, regardless of their molecular structure. In clinical applications, when drugs are administered, if the drug is hydrophobic, some additives or solvents must be used; these may be undesirable for the body. The use of nanofibers as drug carriers is also advantageous in that the drugs can be administered without such additives. Poly(ε-caprolactone) (PCL) was selected as a nanofiber fabrication material in this study and has already been approved for use in clinical settings by the U.S. Food and Drug Administration due to its biodegradable and biocompatible nature. We previously investigated the potential of PCL nanofiber sheets incorporating lenvatinib as a DDS; this DDS exhibited antitumor activity against an in vivo hepatocellular carcinoma model [16]. The study showed that serum lenvatinib concentrations and antitumor activity were maintained regardless of where the sheet was placed, implying that application on the body surface may exert therapeutic benefit in clinical settings.

In this study, we demonstrated that PCL nanofiber sheets incorporating oxaliplatin (oxaliplatin sheets) showed an effective and sustained antitumor effect in a mouse xenograft model of colon and gastric cancers.

## 2. Materials and Methods

### 2.1. Fabrication of Electrospun PCL Nanofiber Sheets

PCL (*Mw*: 80 kDa, 10 *w*/*v*%) and oxaliplatin (5 *w*/*v*%) were dissolved in 1,1,1,3,3,3-hexafluoro-2-propanol. The spinning solution was loaded into a syringe fitted with a 25-gauge needle and electrospun at 25 kV with a 15 cm tip-to-collector distance and a 1.0 mL/h flow rate using a Nanon-01A system (MECC Co., Ltd., Fukuoka, Japan). Fiber morphology was examined by scanning electron microscopy (SEM; SU8230, Hitachi High-Tech, Tokyo, Japan), and fiber diameters were quantified using ImageJ software(version 1.54i).

### 2.2. Drug Release Profile

Nanofiber sheets incorporating 350 μg oxaliplatin (n = 3) were immersed in 5 mL phosphate-buffered saline (PBS) and placed in an incubator at 37 °C while stirring at 100 rpm. At 24 h intervals, the release medium was collected and replaced with 5 mL fresh PBS; sampling continued for 60 days. Oxaliplatin in the supernatants was quantified by measuring absorbance at 250 nm (specific to oxaliplatin), using a NanoDrop (Wilmington, DE, USA) 2000 spectrophotometer (Thermo-Fisher Scientific, Waltham, MA, USA).

### 2.3. Cancer Cell Lines and Reagents

COLO 205 cells, a human colon cancer cell line, were purchased from the RIKEN BioResource Research Center (Ibaraki, Japan), and MKN45-luc cells, a human gastric cancer cell line, were purchased from the Japanese Collection of Research Bioresources Cell Bank (Osaka, Japan). The cells were grown in RPMI 1640 medium (FUJIFILM Wako Pure Chemical Corporation, Osaka, Japan) supplemented with 10% fetal bovine serum (BioWest, Nuaillé, France), 200 units/mL penicillin, 10 mg/mL streptomycin, and 25 mg/mL amphotericin B (SARTORIUS, Göttingen, Germany) and were incubated at 37 °C in a humidified atmosphere containing 5% CO_2_. Oxaliplatin was purchased from Fujifilm Wako Pure Chemical Corporation (Osaka, Japan).

### 2.4. Animals

One hundred seventy BALB/c nude mice (4 weeks old, 15 ± 2 g each) were purchased from The Jackson Laboratory Japan, Inc. (Kanagawa, Japan) and allowed to acclimate for 1 week. Mice were provided with free access to food and water and maintained at 22 °C under a 12/12 h light/dark cycle. All procedures complied with the Animal Welfare Committee guidelines of Kansai Medical University (approval number: 25-041). Every effort was made to minimize the number of animals used and to prevent any pain and suffering.

### 2.5. Treatment of Subcutaneous Tumor Models

A mouse model of colorectal cancer was established based on previous studies [17,18,19], with some modifications. In addition, a mouse model of gastric cancer was also established based on a previous study [20], with some modifications. COLO 205 human colorectal cancer cells (5 × 10^6^/100 µL) or MKN45-luc human gastric cancer cells (5 × 10^6^/100 µL) were injected subcutaneously into the right flanks of mice. Upon reaching a mean tumor volume of approximately 100 mm^3^, mice were randomly assigned to treatment (day 0). Tumor volumes and body weights were measured on days 7, 14, and 21 after allocation. Tumor volume was calculated using the following formula: 1/2 × length × width^2^.

The procedure for implanting the sheets into the mice was as follows. First, each mouse was given a deep anesthetic injection of a mixture of three drugs, which included medetomidine hydrochloride (0.75 mg/kg), midazolam (4 mg/kg), and butorphanol tartrate (5 mg/kg). Then, the skin adjacent to the tumor was incised, and the area beneath the tumor and skin was dissected carefully. Next, one sheet (10 mm × 10 mm) was placed directly beneath the tumor. Finally, the wound was closed and repaired using 2–3 nodal sutures with 5-0 prolene thread.

The antitumor effects of intraperitoneal oxaliplatin and oxaliplatin-incorporated PCL sheets were compared in a subcutaneous tumor model of colorectal cancer. Mice were classified into the following four groups according to the treatment method: the no treatment group (mice were subjected to implantation of a drug-free PCL sheet, n = 10), the intraperitoneal oxaliplatin group (mice were treated with 5 mg/kg/dose oxaliplatin 3 times a week for 3 weeks, n = 9), the 0.5 mg oxaliplatin PCL sheet group (0.5 mg oxaliplatin-incorporated sheets were implanted in mice, 36.7% of the 5 mg/kg dose of intraperitoneal oxaliplatin, n = 10), and the 0.25 mg oxaliplatin PCL sheet group (0.25 mg oxaliplatin-incorporated sheets were implanted in mice, 18.3% of the 5 mg/kg dose of intraperitoneal oxaliplatin, n = 6).

The antitumor effects of sheet implantation position were compared in the colon cancer subcutaneous tumor model. The following four positions of sheet implantation were classified: the control group (no treatment; a drug-free PCL sheet was implanted directly under the subcutaneous tumor, n = 6), the direct group (a 0.5 mg oxaliplatin sheet was implanted directly under the tumor, n = 6), the surrounding group (a 0.5 mg oxaliplatin sheet was divided in half and placed around the tumor, n = 6), and the contralateral group (a 0.5 mg oxaliplatin sheet was placed contralateral to the subcutaneous tumor, n = 6).

In order to compare treatments with intraperitoneal oxaliplatin and oxaliplatin-incorporated PCL sheets, even in a subcutaneous tumor model of gastric cancer, mice were classified into three categories according to the treatment method as follows: the no treatment group (mice were implanted with a drug-free PCL sheet, n = 6), the intraperitoneal oxaliplatin group (mice were treated with 2.5 mg/kg/dose oxaliplatin 3 times a week for 3 weeks, n = 6), and the 0.25 mg oxaliplatin PCL sheet group (mice were implanted with a 0.25 mg oxaliplatin-incorporated sheet, 36.7% of the 2.5 mg/kg dose of intraperitoneal oxaliplatin, n = 6).

### 2.6. Treatment of a Peritoneal Metastasis Model

A model for treating peritoneal metastasis was developed by injecting MKN45-luc cells into the peritoneal cavity. The number of cells administered was adjusted to create the appropriate model. Depending on the treatment method, the mice were randomly classified into a control group (implanted with a drug-free PCL sheet, n = 13) and a 0.25 mg oxaliplatin group (implanted with a 0.25 mg oxaliplatin-incorporated PCL sheet, n = 13). For cell administration and sheet implantation, the mice were deeply anesthetized using a mixture of medetomidine hydrochloride (0.75 mg/kg), midazolam (4 mg/kg), and butorphanol tartrate (5 mg/kg). MKN45-luc cells (2 × 10^7^/500 µL) were injected into the peritoneal cavity, and drug-free or oxaliplatin sheets were implanted simultaneously. We set day 0 as the day of sheet implantation and prospectively recorded overall survival.

### 2.7. Histopathological Examination

Subcutaneous xenograft tumors and livers were harvested on day 21 of treatment. Tumor fragments were fixed in 10% formalin and embedded in paraffin. Sections (approximately 4 μm thick) were prepared from each block, hematoxylin and eosin (H&E) staining was performed on the tumors and livers, and Ki-67 immunostaining was performed on the tumors to assess the degree of proliferative activity of tumor cells. Anti-human Ki-67 mouse monoclonal antibodies (clone: MIB1; cat. no. M7240; DAKO-Agilent Technologies, Santa Clara, CA, USA) were diluted 1:500 and stained using an automated staining machine (BOND MAX; Leica Biosystems Melbourne Pty Ltd., Melbourne, Australia). The Ki-67 labeling index (Ki-67 LI) and mitotic index (MI) were measured in the tumors. The Ki-67 LI was measured using a microscope (BX-4310N; ERMA, Tokyo, Japan) with a cell calculator (model F410N; ERMA, Tokyo, Japan) at a site with a relatively high number of positive cells (hot spot) in the tumor foci that avoided necrotic areas in Ki-67 immunostaining. The percentage of Ki-67-positive cells in 1000 tumor cells was measured under direct observation with a microscope (BX-43; OLYMPUS Corporation, Tokyo, Japan) at 400× magnification. MI was measured as the percentage of morphologically dividing cells in 1000 tumor cells under direct observation at 400× magnification after H&E staining.

### 2.8. Measurement of Oxaliplatin Concentrations in Blood

Oxaliplatin concentrations in blood were measured using a method developed based on previous studies [21,22,23], with some modifications. Blood samples were collected 1, 3, 7, 14, and 21 days after sheet implantation or 20, 40, 60, 80, and 120 min after intraperitoneal oxaliplatin administration. The procedure for blood collection was as follows. After deep anesthesia via intraperitoneal injection of medetomidine (0.75 mg/kg), midazolam (4 mg/kg), and butorphanol (5 mg/kg), mice were positioned supine, and approximately 1 mL of blood was obtained from the inferior vena cava using a 26 G needle. Euthanasia was completed by cervical dislocation. Blood samples were placed in EDTA-2Na-treated tubes. The tubes were then centrifuged at 2000× *g* for 15 min, and the supernatant was collected and stored at −80 °C until the blood concentration was determined. To extract oxaliplatin from plasma samples, 30 μL of acetone was added to 30 μL of plasma, and samples were allowed to stand on ice for 5 min. The samples were then centrifuged at 4 °C and 15,000 rpm for 5 min. The supernatant was analyzed using liquid chromatography and mass spectrometry (LC-MS/MS). LC was performed using a Prominence HPLC system (Shimadzu Corporation, Kyoto, Japan), and the chromatography system was operated using Analyst (version 1.7.1; SCIEX, Framingham, MA, USA). Chromatographic separation was performed at 40 °C using a Hypercarb Porous Graphitic Carbon HPLC columns (Thermo Fisher Scientific; 3 µm particle size, 2.1 mm diameter, 150 mm long). The mobile phase consisted of two solvents (solvent A: 0.1% trifluoroacetic acid [FUJIFILM Wako Pure Chemical Corporation] in ultrapure water [FUJIFILM Wako Pure Chemical Corporation]; solvent B: 0.1% trifluoroacetic acid in methanol [FUJIFILM Wako Pure Chemical Corporation]). Then, 15 µL of the sample was injected into the column and separated at a flow rate of 0.2 mL/min for 20 min using the following program: a 5 min isocratic flow of 5% solvent B, a 5 min linear gradient flow from 5% to 100% solvent B, and a 10 min isocratic flow of 100% solvent B. After this program, the column was equilibrated with 5% solvent B for 5 min. The eluate was introduced into an API 3200 mass spectrometer (SCIEX). The retention time of oxaliplatin was about 10.6 min. The oxaliplatin concentration in plasma was determined by counting the peak area at the desired retention time; each sample was measured twice, and the average value was determined. Because acetone extraction recovery was equivalent across serum samples, between-sample comparisons used the detected oxaliplatin as a relative measure of drug level.

### 2.9. Statistical Analysis

Data are presented as means ± standard errors. Comparisons of tumor volumes and body weights between groups were performed with a generalized linear mixed model. Fixed effects included colorectal cancer treatment, attachment location of the sheet, gastric cancer treatment, and the observation period after treatment. Time was coded as weeks from the time of treatment initiation. We included the mice as a random effect. We then evaluated the results by multiple comparisons using the Tukey–Kramer multiple comparison test. Ki-67 LI and MI were evaluated by one-way analysis of variance between treatment groups and by multiple comparisons with the Tukey–Kramer test. Overall survival was analyzed with the Kaplan–Meier method and compared by log-rank testing; results with a *p* value less than 0.05 were considered statistically significant. Statistical analyses were performed using R software (version 4.1.2; R Foundation for Statistical Computing, Vienna, Austria) with the lme4 package (version 1.1.36) and lmerTest package (version 3.1.3) and JMP Pro (version 17.0.0; SAS Institute Inc., Cary, NC, USA).

## 3. Results

### 3.1. Sustainable Oxaliplatin Release from PCL Sheets

**Figure 1:**
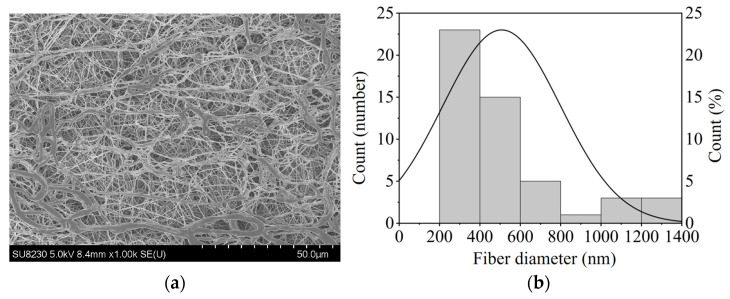
(**a**) The morphology of PCL nanofiber sheets incorporated with oxaliplatin, imaged using SEM; (**b**) The fiber diameter of the oxaliplatin-incorporated PCL nanofibers (n = 50).

Oxaliplatin-incorporated nanofiber sheets were fabricated with PCL by electrospinning under carefully optimized conditions [15]. PCL is widely used in biomaterials as a biodegradable polymer and does not need to be removed after the nanofiber sheet is implanted in the body and treatment is completed. It is also very flexible, making it easy to place along the affected area. Based on a previous report [15], we carefully selected the PCL concentration because solution viscosity is a primary determinant of electrospinnability. The nanofiber morphology was observed by scanning electron microscopy, and the diameter of nanofibers with oxaliplatin was 507.5 nm (n = 50) with highly uniformed morphology (Figure 1).

**Figure 2:**
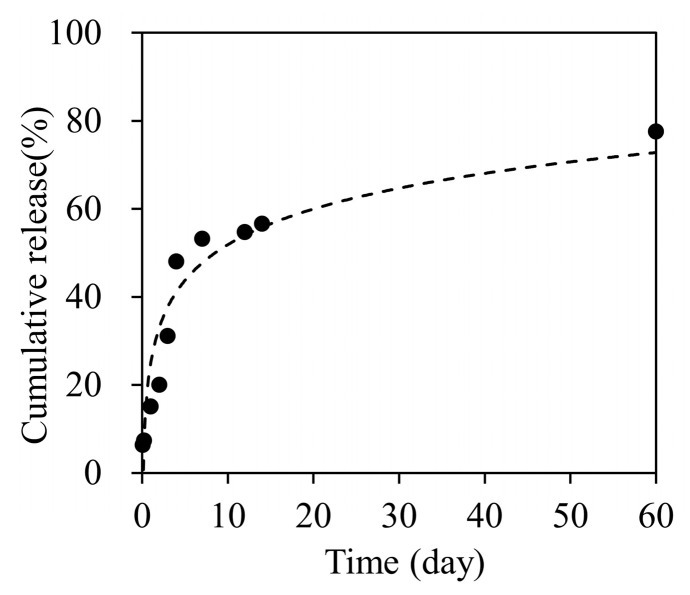
Cumulative oxaliplatin release profile for 60 days.

The nanofiber sheets released oxaliplatin sustainedly over 60 days (Figure 2). The amounts of oxaliplatin released at 7, 14, and 60 days were 53.2%, 56.6%, and 77.6%, respectively. The results showed that approximately 0.33 mg oxaliplatin was released over 21 days from the sheet containing 0.5 mg of the drug. This corresponded to approximately 36.7% of the oxaliplatin administered by intraperitoneal injection of 5.0 mg/kg/dose (0.9 mg/9 doses over 21 days) in mice (when assuming a body weight of 20 g).

### 3.2. Antitumor Effects of Oxaliplatin Sheets in a Human Colon Cancer Subcutaneous Xenograft Tumor Model

**Figure 3:**
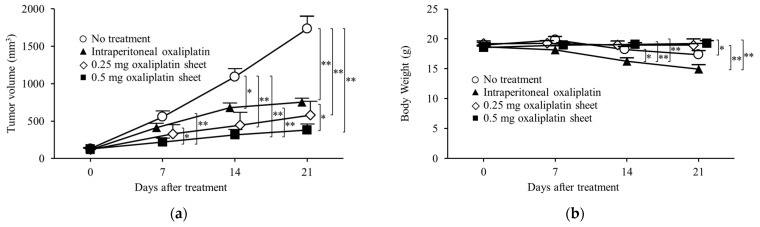
Antitumor effects of oxaliplatin sheets in a human colorectal cancer subcutaneous xenograft tumor model. CLOL 205 human colon cancer cells (5 × 10^6^) were transplanted subcutaneously into BALB/c nude mice. Mice were classified into four groups based on treatment: no treatment group (open circle; n = 10), intraperitoneal group (closed triangle; n = 9), 0.25 mg oxaliplatin sheet group (open diamond; n = 6), and 0.5 mg oxaliplatin sheet group (closed square; n = 10). (**a**) Tumor volumes; (**b**) Body weights. * *p* < 0.05, ** *p* < 0.01.

In a subcutaneous tumor model of colon cancer, the antitumor effects of oxaliplatin were compared between the intraperitoneal oxaliplatin group and the oxaliplatin sheet groups (Figure 3a). The intraperitoneal group showed significant tumor growth suppression compared with the no treatment group on day 14 (tumor volumes: intraperitoneal group, 679 ± 65 mm^3^ versus no treatment group, 1093 ± 111 mm^3^; *p* = 0.014) and day 21 (intraperitoneal group, 756 ± 52 mm^3^ versus no treatment group, 1735 ± 171 mm^3^; *p* < 0.001). The 0.5 mg oxaliplatin sheet group showed significant tumor growth suppression compared with the no treatment group on day 7 (tumor volume: 0.5 mg oxaliplatin sheet group, 222 ± 53 mm^3^ versus no treatment group, 558 ± 76 mm^3^; *p* < 0.001), day 14 (0.5 mg oxaliplatin group, 317 ± 69 mm^3^ versus no treatment group, 1093 ± 111 mm^3^; *p* < 0.001), and day 21 (0.5 mg oxaliplatin group, 383 ± 77 mm^3^ versus no treatment group, 1735 ± 171 mm^3^; *p* < 0.001). Furthermore, the 0.5 mg oxaliplatin sheet group showed significant tumor growth suppression compared with the intraperitoneal group on day 7 (tumor volumes: 0.5 mg oxaliplatin group, 222 ± 53 mm^3^ versus intraperitoneal group, 416 ± 55 mm^3^; *p* = 0.021), day 14 (0.5 mg oxaliplatin group, 317 ± 69 mm^3^ versus intraperitoneal group, 679 ± 65 mm^3^; *p* = 0.004), and day 21 (0.5 mg oxaliplatin group, 383 ± 77 mm^3^ versus intraperitoneal group, 756 ± 52 mm^3^; *p* = 0.021). The 0.25 mg oxaliplatin sheet group showed significant tumor growth suppression compared with the no treatment group on day 14 (tumor volumes: 0.25 mg oxaliplatin group, 447 ± 171 mm^3^ versus intraperitoneal group, 1093 ± 111 mm^3^; *p* < 0.001) and day 21 (0.25 mg oxaliplatin group, 581 ± 183 mm^3^ versus intraperitoneal group, 1735 ± 171 mm^3^; *p* < 0.001). The 0.5 mg oxaliplatin sheet group showed a volume-dependent antitumor effect compared with the 0.25 mg oxaliplatin sheet group, although there was no statistically significant difference.

In our analysis of weight loss as a side effect of oxaliplatin, the intraperitoneal group lost more weight than the 0.5 mg oxaliplatin sheet group on day 14 (weight: intraperitoneal group, 16.24 ± 0.58 g versus 0.5 mg oxaliplatin sheet group, 19.06 ± 0.32 g; *p* < 0.001) and day 21 (intraperitoneal group, 14.91 ± 0.76 g versus 0.5 mg oxaliplatin group, 19.26 ± 0.42 g; *p* < 0.001; Figure 3b). The intraperitoneal group also showed weight loss compared with the 0.25 mg oxaliplatin sheet group on day 14 (weight: intraperitoneal group, 16.24 ± 0.58 g versus 0.25 mg oxaliplatin sheet group, 18.99 ± 0.68 g; *p* = 0.012) and day 21 (intraperitoneal group, 14.91 ± 0.76 g versus 0.25 mg oxaliplatin sheet group, 18.94 ± 1.09 g; *p* = 0.004). The no treatment group showed statistically significant weight loss compared with the intraperitoneal group on day 14 (weight: no treatment group, 18.23 ± 0.51 g versus intraperitoneal group, 16.24 ± 0.58 g; *p* = 0.046) and compared with the 0.5 mg oxaliplatin sheet group on day 21 (weight: no treatment group, 17.37 ± 0.62 g versus 0.5 mg oxaliplatin sheet group, 19.26 ± 0.42 g; *p* = 0.044).

### 3.3. Pathological Evaluation of Antitumor Effects by Oxaliplatin in Subcutaneous Tumors and Adverse Effects of Oxaliplatin in the Liver

Subcutaneous tumors and livers were collected 21 days after treatment in the no treatment, intraperitoneal, and 0.5 mg oxaliplatin sheet groups. Subcutaneous tumors were then evaluated by H&E staining and Ki-67 immunostaining, and livers were evaluated by H&E staining.

**Figure 4:**
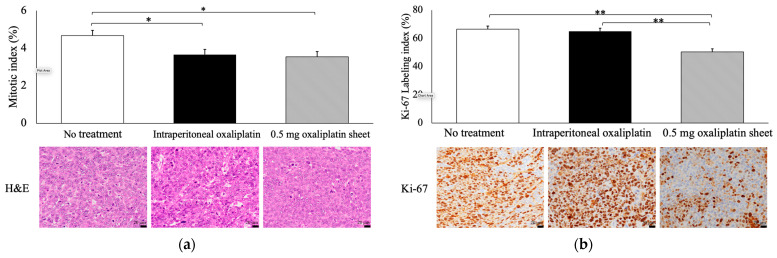
Pathological evaluation of antitumor effects by oxaliplatin in subcutaneous tumors. Tumors were collected on day 21 after sheet implantation or the first dose of oxaliplatin. Formalin-fixed, paraffin-embedded tumor sections were then stained with H&E (C; 400×, bar = 20 μm) and Ki-67 (D; 400×, bar = 20 μm). (**a**) The mitotic index (%) was measured as the percentage of morphologically mitotic cells in 1000 tumor cells in H&E staining. (**b**) The Ki-67 labeling index (%) was measured as the percentage of Ki-67-positive cells in 1000 tumor cells in Ki-67 immunostaining. Data are presented as means ± standard errors (n = 7 for each group). * *p* < 0.05, ** *p* < 0.01.

Both the 0.5 mg oxaliplatin sheet and intraperitoneal groups showed significantly lower MIs than the no treatment group (0.5 mg oxaliplatin sheet group, 3.54% and intraperitoneal group, 3.66% versus no treatment group, 4.67%; *p* = 0.023, *p* = 0.042, respectively; Figure 4a). Moreover, the 0.5 mg oxaliplatin sheet group showed a significantly lower Ki-67 LI than the intraperitoneal oxaliplatin group and the no treatment group (0.5 mg oxaliplatin sheet group, 50.4% versus intraperitoneal group, 65.0% and no treatment group, 66.5%; *p* < 0.001, *p* < 0.001, respectively; Figure 4b).

**Figure 5:**
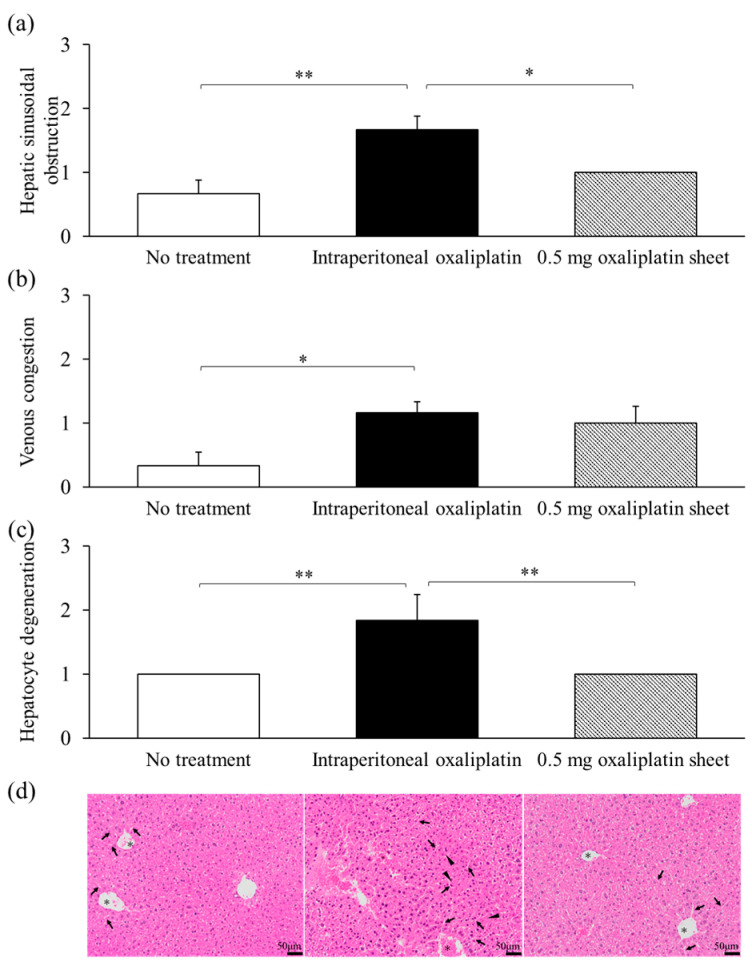
Pathological evaluation of adverse events induced by oxaliplatin in the liver. Livers were collected on day 21 after sheet implantation or after receiving the first dose of oxaliplatin. Sections of the liver were treated with formalin and paraffin and then stained with H&E; the arrows indicate sinusoidal dilatation, the triangles indicate hepatocyte degeneration, and the asterisks indicate congestion (**d**); 400×, bar = 50 μm) for evaluation. The degrees of hepatic sinusoidal obstruction (**a**), venous congestion (**b**), and hepatocyte degeneration (**c**) were scored and assessed. The scores were as follows: 0 for no significant change, 1 for slightly increased, 2 for relatively increased, and 3 for significantly increased. Data are presented as means ± standard errors (n = 6 for each group). * *p* < 0.05, ** *p* < 0.01.

Hepatic sinusoidal obstruction was significantly increased in the intraperitoneal oxaliplatin group compared with the no treatment and 0.5 mg oxaliplatin sheet groups (intraperitoneal group, 1.67 versus no treatment group, 0.67 and 0.5 mg oxaliplatin sheet group, 1.0; *p* = 0.003, *p* = 0.038, respectively; Figure 5a,d). Moreover, congestion was significantly higher in the intraperitoneal oxaliplatin group than in the no treatment group (intraperitoneal group, 1.17 versus no treatment group, 0.33; *p* = 0.038; Figure 5b,d). Hepatocellular degeneration was also significantly increased in the intraperitoneal oxaliplatin group compared with the no treatment and 0.5 mg oxaliplatin sheet groups (intraperitoneal group, 1.83 versus no treatment group, 1.0 and 0.5 mg oxaliplatin sheet group, 1.0; *p* < 0.001; Figure 5c,d).

### 3.4. Antitumor Effects of Oxaliplatin Sheets in Different Implantation Positions

**Figure 6:**
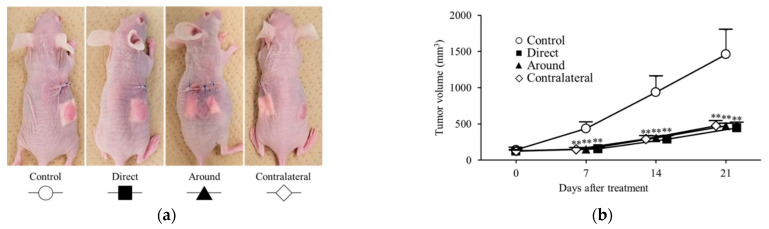
Antitumor effects of oxaliplatin sheets in different implantation positions. Mouse models of human colorectal cancer xenograft tumors were classified into four groups based on the position of the sheet during implantation. The groups used were as follows: control group (represented by open circles; n = 6), direct group (represented by closed squares; n = 6), peripheral group (represented by closed triangles; n = 6), and contralateral group (represented by open diamonds; n = 6). (**a**) Diagram depicting the position of sheet implantation in mice; (**b**) Tumor volumes. ** *p* < 0.01.

Subcutaneous xenograft mouse models of colon cancer were classified into the direct, around, contralateral, and control groups according to the implantation position of the oxaliplatin PCL sheet (Figure 6a). On day 7 of treatment, a significantly greater antitumor effect on tumor volume was observed in the direct group (156 ± 28 mm^3^; *p* = 0.004), the around group (148 ± 17 mm^3^; *p* = 0.003), and the contralateral group (149 ± 21 mm^3^; *p* = 0.003) compared with that in the control group (435 ± 92 mm^3^).

Similarly, on day 14 of treatment, a significantly greater antitumor effect on tumor volume was observed in the direct group (287 ± 50 mm^3^; *p* = 0.004), the around group (305 ± 25 mm^3^; *p* = 0.005), and the contralateral group (292 ± 45 mm^3^; *p* = 0.004) compared with that in the control group (937 ± 221 mm^3^). Finally, on day 21 of treatment, significant tumor growth suppression was observed in the direct group (443 ± 72 mm^3^; *p* = 0.004), the around group (472 ± 32 mm^3^; *p* = 0.004), and the contralateral group (473 ± 69 mm^3^; *p* = 0.005; Figure 6b) compared with that in the control group (1459 ± 342 mm^3^). On treatment days 7, 14, and 21, tumor volumes did not differ significantly across the three sheet-placement sites.

### 3.5. Trends in Blood Oxaliplatin Concentrations

**Figure 7:**
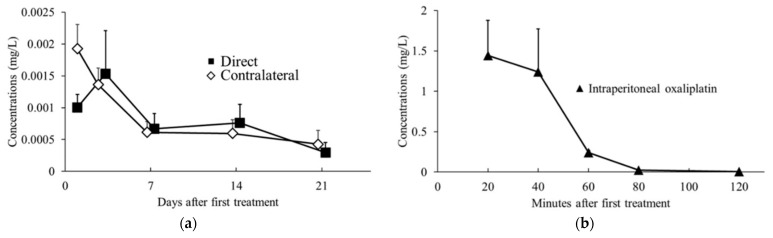
Trends in blood oxaliplatin concentrations. Blood samples were collected 1, 3, 7, 14, and 21 days after sheet implantation or 20, 40, 60, 80, and 120 min after intraperitoneal oxaliplatin administration and analyzed using LC-MS/MS. (**a**) Changes in concentrations after intraperitoneal administration of oxaliplatin (closed triangles; n = 4–5 for per time point); (**b**) Changes in concentrations after subcutaneous implantation of oxaliplatin sheets (direct group, closed squares, n = 4–6 for each day; contralateral group, open diamonds, n = 5–6 for each day).

Relative plasma oxaliplatin concentrations were measured using LC-MS/MS and compared between the intraperitoneal oxaliplatin, oxaliplatin sheet direct implantation, and oxaliplatin sheet contralateral implantation groups. The plasma concentrations in the oxaliplatin sheet direct implantation group and the oxaliplatin sheet contralateral implantation group decreased slowly over time (Figure 7a). In the intraperitoneal oxaliplatin group, the plasma concentration decreased rapidly after administration, and at 80 and 120 min after administration, the plasma concentration was very low (Figure 7b). The plasma concentrations in the oxaliplatin sheet direct implantation group and the oxaliplatin sheet contralateral implantation group remained lower than those at 120 min after intraperitoneal administration for the entire period.

### 3.6. Oxaliplatin Sheets Showed Antitumor Effects in a Human Gastric Cancer Subcutaneous Xenograft Tumor Model

**Figure 8:**
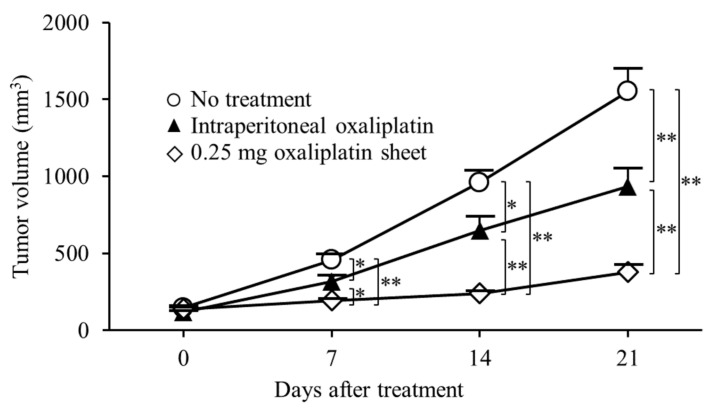
Antitumor effects of oxaliplatin sheets in a human subcutaneous tumor model of gastric cancer. MKN45-luc human gastric carcinoma cells (5 × 10^6^) were transplanted subcutaneously into BALB/c nude mice. Mice were classified into three groups based on treatment: no treatment group (open circles; n = 6), intraperitoneal group (closed triangles; n = 6), and 0.25 mg oxaliplatin sheet group (open diamonds; n = 6). Data are presented as means ± standard errors. * *p* < 0.05, ** *p* < 0.01.

The antitumor effects of oxaliplatin-incorporated PCL nanofiber sheets were also shown in a human subcutaneous tumor model of gastric cancer. In this subcutaneous tumor model, we compared the antitumor efficacy of oxaliplatin PCL sheets with intraperitoneal oxaliplatin, similar to the subcutaneous colon cancer model (Figure 8). The 0.25 mg oxaliplatin sheet group showed significant tumor growth suppression compared with the no treatment group on day 7 (tumor volumes: 0.25 mg oxaliplatin sheet group, 192 ± 13 mm^3^ versus no treatment group, 455 ± 38 mm^3^; *p* < 0.001), day 14 (0.25 mg oxaliplatin sheet group, 238 ± 18 mm^3^ versus no treatment group, 960 ± 76 mm^3^; *p* < 0.001), and day 21 (0.25 mg oxaliplatin sheet group, 376 ± 49 mm^3^ versus no treatment group, 1552 ± 151 mm^3^; *p* < 0.001). The intraperitoneal group showed significant tumor growth suppression compared with the no treatment group on day 7 (tumor volumes: intraperitoneal group, 317 ± 35 mm^3^ versus no treatment group, 455 ± 38 mm^3^; *p* = 0.017), day 14 (intraperitoneal group, 646 ± 90 mm^3^ versus no treatment group, 960 ± 76 mm^3^; *p* = 0.015), and day 21 (intraperitoneal group, 933 ± 118 mm^3^ versus no treatment group, 1552 ± 151 mm^3^; *p* = 0.005). Furthermore, the 0.5 mg oxaliplatin sheet showed significant tumor growth suppression compared with the intraperitoneal group on day 7 (tumor volumes: 0.25 mg oxaliplatin sheet group, 192 ± 13 mm^3^ versus intraperitoneal group, 317 ± 35 mm^3^; *p* = 0.031), day 14 (0.25 mg oxaliplatin sheet group, 238 ± 18 mm^3^ versus intraperitoneal group, 646 ± 90 mm^3^; *p* = 0.002), and day 21 (0.25 mg oxaliplatin sheet group, 376 ± 49 mm^3^ versus intraperitoneal group, 933 ± 118 mm^3^; *p* = 0.009).

### 3.7. Effects of Oxaliplatin Sheets in a Mouse Model of Peritoneal Seeding of Gastric Cancer Cells

**Figure 9:**
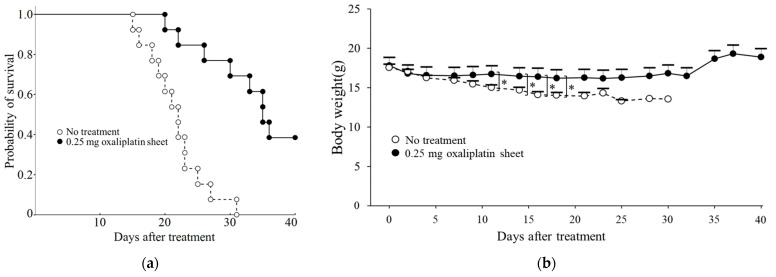
Effects of oxaliplatin sheets on survival in a mouse model of peritoneal dissemination of gastric cancer. BALB/c nude mice were injected intraperitoneally with MKN45-luc cells, and at the same time, 0.25 mg of oxaliplatin sheets or drug-free sheets were implanted subcutaneously in mice. (**a**) Survival rates are shown for the control group (open circles: n = 13) and the 0.25 mg oxaliplatin sheet group (closed circles: n = 13). Kaplan–Meier survival analysis was performed with log-rank tests; (**b**) Body weights in the control group (open circles: days 0–14, n = 13; day 16, n = 12; day 18, n = 10; day 21, n = 7; day 23, n = 3; day 25, n = 2; days 28–30, n = 1) and the 0.25 mg oxaliplatin sheet group (closed circles: days 0–18, n = 13; day 21, n = 12; days 23–25, n = 11; day 28, n = 10; days 30–32, n = 9; days 37–40, n = 5). * *p* < 0.05.

Oxaliplatin PCL sheets improved survival in a mouse model of peritoneal dissemination of human gastric cancer. Compared with the control group, mice receiving the 0.25 mg oxaliplatin sheet had a significantly prolonged overall survival (*p* < 0.001; Figure 9a). A small amount of bloody ascites was observed in the control group but not in the 0.25 mg oxaliplatin sheet group. On treatment day 11, although no mice had died in either group, the controls exhibited significant weight loss (Figure 9b).

## 4. Discussion

In this study, PCL nanofiber sheets incorporated with oxaliplatin, which is a third-generation platinum compound, functioned as an effective controlled-release DDS, producing antitumor efficacy with a favorable safety profile. Despite releasing a lower cumulative amount of oxaliplatin than total intraperitoneal dosing, the sheets sustained a steady release profile, significantly suppressed subcutaneous gastrointestinal tumor growth, and improved survival in a gastric cancer peritoneal metastasis model (Figure 3a, Figure 8 and Figure 9). In terms of safety, the oxaliplatin-sheet group exhibited no significant body-weight loss (Figure 3b and Figure 9b) and less hepatic injury compared with the group administered intraperitoneal oxaliplatin (Figure 5, Supplementary Table S1).

For in vitro release of oxaliplatin-loaded nanofiber sheets, the drug was released sustainably (Figure 2). Drug release occurred mainly by slow diffusion through the hydrophobic PCL matrix, providing sustained, low-concentration exposure and reducing the initial burst [24,25]. By contrast, with intraperitoneal administration, oxaliplatin showed a rapid decline in blood levels due to its short plasma half-life, a transient peak followed by quick troughs (Figure 7b). The sustained release of oxaliplatin from the oxaliplatin sheet maintained lower concentrations of oxaliplatin in the oxaliplatin sheet-implanted group because oxaliplatin did not accumulate in the blood due to the short half-life of oxaliplatin. In an in vitro study, the half-maximal inhibitory concentration of oxaliplatin tended to decrease as the exposure time increased in COLO 205 human colon cancer cells [26]. Notably, continuous administration of oxaliplatin is reported to increase the area under the concentration-time curve (AUC) of free platinum, which is supposed to have an antitumor effect, although the AUC of total platinum is similar [27]. Despite not measuring total or free platinum, the data support the interpretation that free platinum is the principal active form driving antitumor effects via DNA–platinum adduct formation, inhibition of DNA replication/transcription, and sustained suppression of DNA synthesis, ultimately reducing cell-cycle progression as reflected in lower Ki-67 and mitotic indices (Figure 4) [27,28,29,30]. Moreover, even with a lower total dose than intraperitoneal administration, the oxaliplatin sheets suppressed gastric and colorectal tumor growth (Figure 3a and Figure 8) and reduced proliferative activity (Figure 4). Taken together, these findings demonstrated the superiority of PCL sheets over conventional intraperitoneal administration.

Electrospun nanofibers combine a very high surface-area-to-volume ratio with controllable porosity and tunable drug-release kinetics, making them attractive drug-delivery platforms in medicine [25]. A prior study using colorectal cell models (CT26), electrospun poly(L-lactide) (PLLA) nanofibers coloaded with oxaliplatin, and 5-fluorouracil showed that direct on-tumor implantation resulted in markedly suppressed tumor growth and prolonged survival compared with intravenous administration [31]. Oxaliplatin was also co-encapsulated with the metabolic modulator dichloroacetate (DCA) in PLLA nanofibers, and implantation of these dual-drug multilayer mats on the resection margin of murine cervical carcinoma produced superior 30-day anti-recurrence efficacy with reduced systemic toxicity [32]. While the prior studies used doses equivalent to existing treatments [31,32], our results are consistent with theirs and extend them by showing that single-agent oxaliplatin delivered via PCL nanofiber sheets achieves robust antitumor activity at a dose lower than the intraperitoneal regimen. Moreover, the PCL nanofiber sheets provided a sustained, low-amplitude release with a reduced initial burst relative to PLLA, which likely contributed to the efficacy observed in our models.

The antitumor effect was equivalent regardless of the position of the sheet during implantation (Figure 6). Moreover, in the peritoneal dissemination model, prolonged survival compared with the untreated group was also shown (Figure 9), indicating the antitumor effect on subcutaneous tumors and peritoneal dissemination nodules that were distant from sheet implantation. These results could be attributed to the slow release of the drug from the sheet, which was absorbed by the capillaries in the skin or peritoneum, transported through the general circulation, and diffused throughout the body. Therefore, we concluded that the drug circulated through the body and was absorbed into the tumor via the tumor’s capillaries, resulting in an antitumor effect. The PCL nanofiber sheets used in our study were thin, soft sheets that could be easily cut into the required size and shape [33]. In clinical settings, direct on-tumor placement is unlikely to be feasible in the stomach and colorectum because these organs are hollow; thus, we suggest that subcutaneous placement could still provide therapeutically relevant exposure.

One common adverse event associated with oxaliplatin treatment is anorexia due to fatigue. In this study, no treatment resulted in weight loss, which may have been due to cancer cachexia (Figure 3b and Figure 9b). Additionally, weight loss was reduced in the oxaliplatin sheet group compared with the intraperitoneal oxaliplatin group (Figure 3b). We also examined liver damage caused by SOS [8] induced by oxaliplatin administration. We found that intraperitoneal oxaliplatin administration caused sinusoidal obstruction, venous stasis, and degeneration of hepatocytes in the liver (Figure 5). Additionally, elevated liver enzymes were observed in blood tests (Supplemental Table S1), but these findings were suppressed in the oxaliplatin sheet. The oxaliplatin sheet also reduced the total dose of oxaliplatin, which may have resulted in a decrease in these adverse events. In addition to weight loss and liver injury, the oxaliplatin sheet may reduce the incidence of volume toxicities, such as vomiting, diarrhea, pancytopenia, and peripheral neuropathy.

PCL sheets incorporated with anticancer drugs are an effective controlled-release DDS that delivers antitumor effects while minimizing adverse events. It allows for stable, sustained, low-concentration administration of anticancer drugs and reduces the total dosage required. There have been fewer studies on controlled-release DDS that can continuously administer anticancer drugs compared with targeted DDS aiming for EPR effects [34]. This study is valuable for advancing the clinical application of controlled-release DDS. In addition, circadian rhythms have attracted attention for their potential to reduce toxicity and increase antitumor effects through optimal timing of anticancer drug administration [35,36]. Various methods for remote control of DDS using ultrasound [37], magnetic fields [38], thermal neutrons [15], and near-infrared light [39] have been reported. If methods to control the release of drugs from the PCL sheet by external manipulation are developed, PCL sheets incorporating anticancer drugs will be clinically applied as a reservoir drug that can release drugs into the body as needed [30].

Our study has several limitations. First, adverse events were monitored only for around 3 weeks after implantation; thus, longer follow-up is needed to define long-term safety. Second, in vivo degradation was not characterized, which is important for clinical dwell-time and dosing intervals. Third, patient-specific tumor microenvironment factors were beyond the scope of this work; xenograft models limit assessment of host immune and stromal interactions relative to syngeneic or patient-derived models. Future studies will systematically evaluate long-term safety and in vivo biodegradation and test PCL sheets loaded with additional anticancer agents, both single agents and combinations, including immune checkpoint inhibitors and molecularly targeted therapies.

## 5. Conclusions

In this study, we found that PCL sheets incorporating oxaliplatin exhibited practical antitumor effects in colon and gastric cancer mouse models. PCL sheets incorporating anticancer drugs may be effective as controlled-release DDSs that can continuously release anticancer drugs over an extended period.

## Data Availability

Data supporting the results of this study are available upon reasonable request from the corresponding authors.

## Supplementary Materials

The following supporting information can be downloaded at https://www.mdpi.com/article/10.3390/nano15191524/s1: Table S1. Blood test evaluation of liver damage for each treatment.

## Abbreviations

The following abbreviations are used in this manuscript:

DDS	Drug delivery systems
PCL	Poly(ε-caprolactone)
SOS	Sinusoidal obstructive syndrome
EPR	Enhanced permeability and retention
PBS	Phosphate-buffered saline
H&E	Hematoxylin and eosin
Ki-67 LI	Ki-67 labeling index
MI	Mitotic index
AUC	Area under the concentration-time curve
PLLA	poly(L-lactide)
