# Supplementary material for: Efficacy of Nanofiber Sheets Incorporating Oxaliplatin in Gastrointestinal Cancer Xenograft Models
Source: Nanomaterials (Basel). 2025 Oct 5;15(19):1524. doi: 10.3390/nano15191524 (PMC12526398; doi:10.3390/nano15191524)
Supplement: Supplementary file 1 [file nanomaterials-15-01524-s001.zip › nanomaterials-3834374-supplementary.pdf]

Supplemental Table 1. Blood test evaluation of liver damage for each treatment

Group	Aspartate transaminase (IU/L)	Alanine transaminase (IU/L)	Lactat dehydrogenase (IU/L)
No treatment	156 ± 20	43 ± 9	881 ± 89
Intraperitoneal oxaliplatin	1585 ± 1199	921 ± 815	2515 ± 1376
0.5mg oxaliplatin sheet	121 ± 18	29 ± 2	821 ± 94
